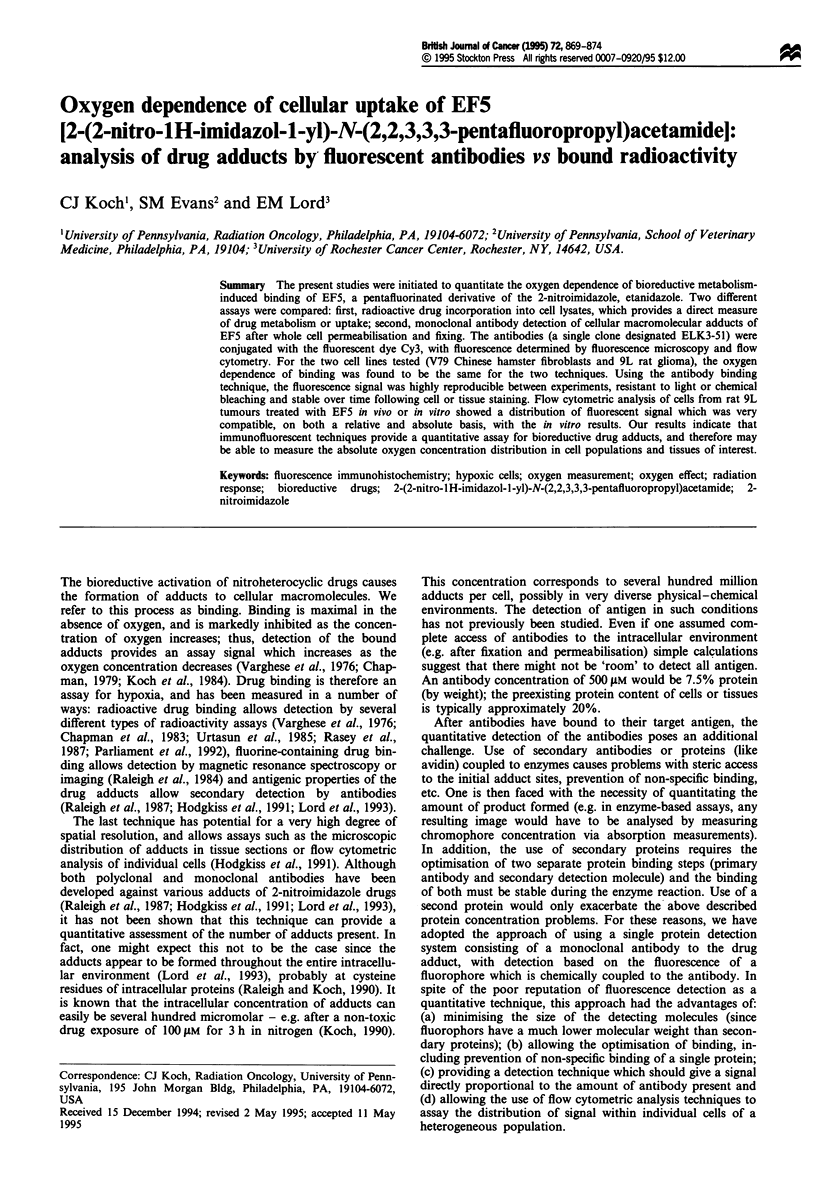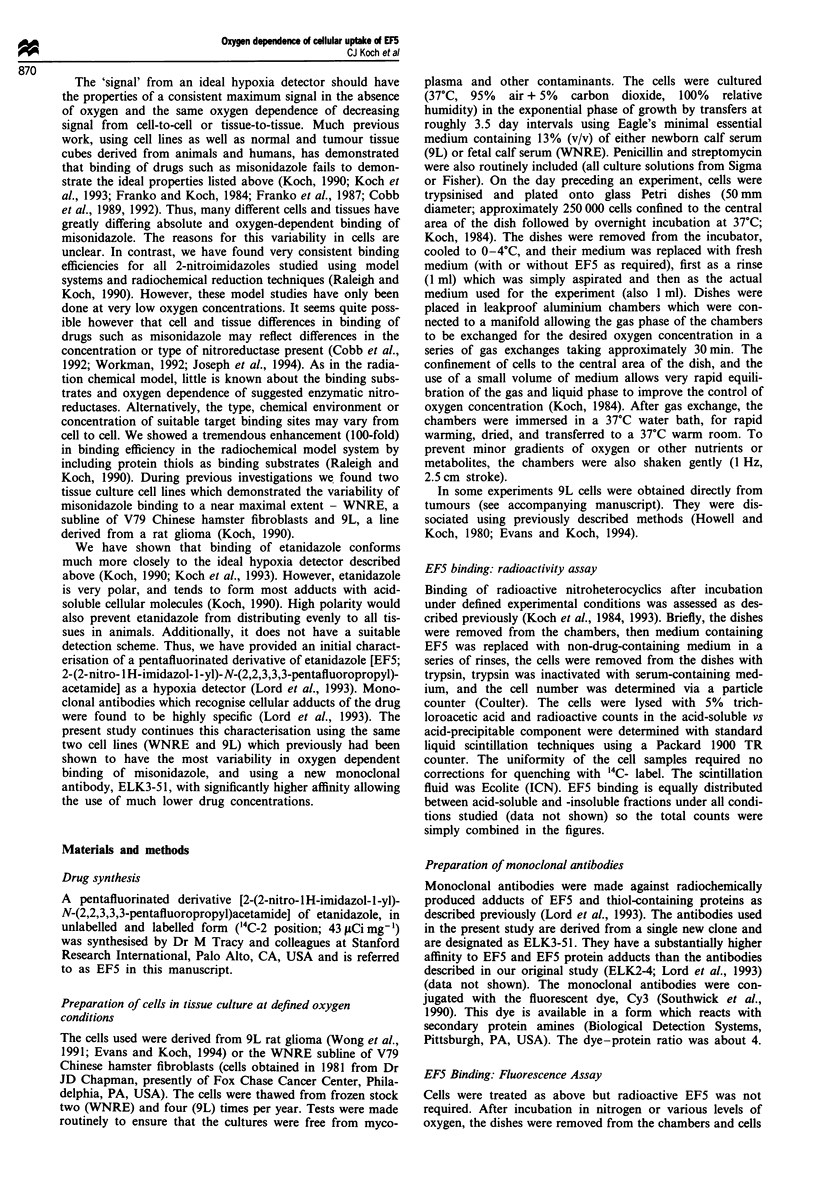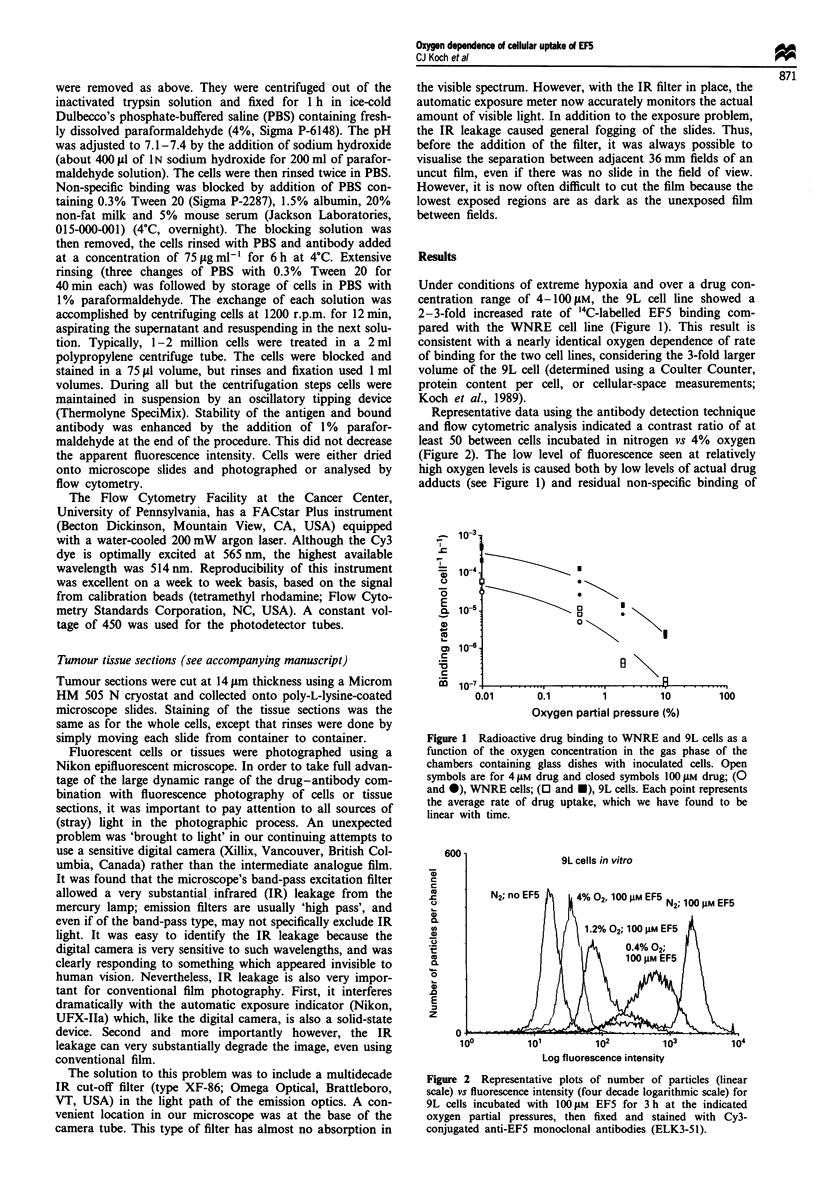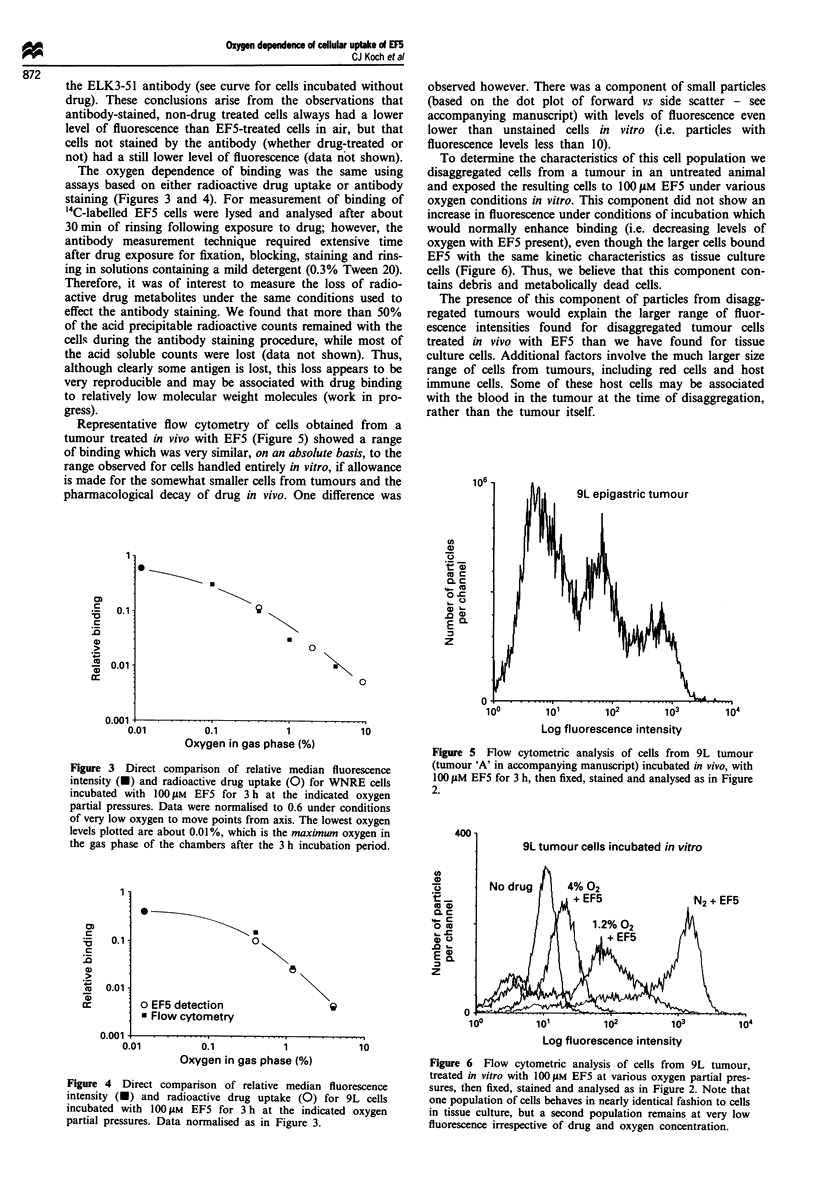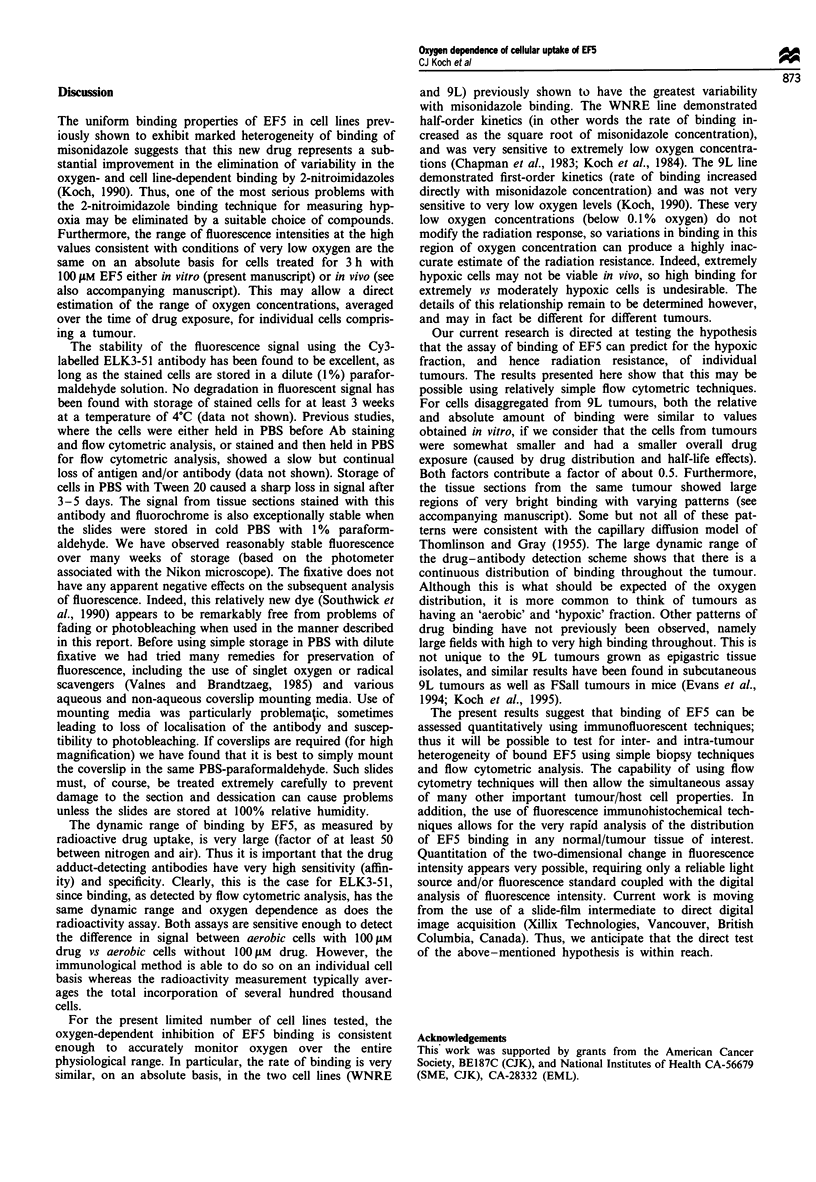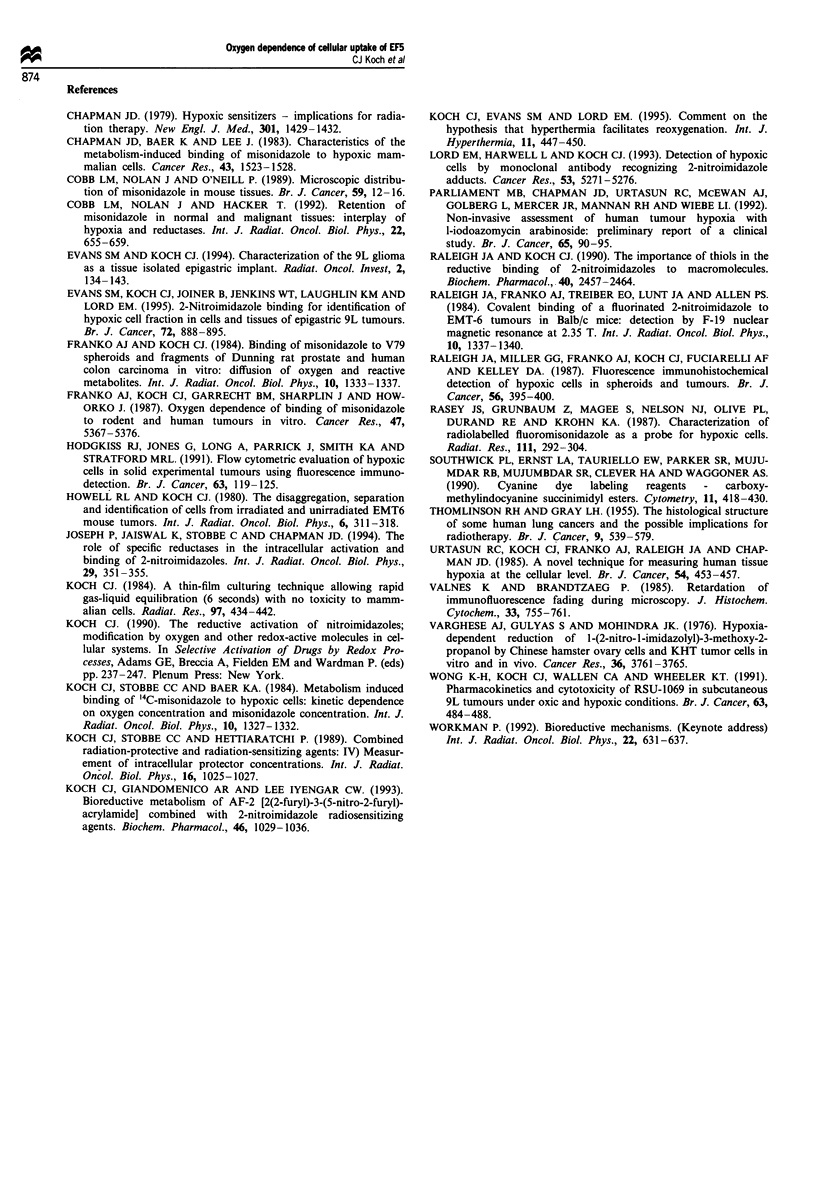# Oxygen dependence of cellular uptake of EF5 [2-(2-nitro-1H-imidazol-1-yl)-N-(2,2,3,3,3-pentafluoropropyl)a cet amide] : analysis of drug adducts by fluorescent antibodies vs bound radioactivity.

**DOI:** 10.1038/bjc.1995.426

**Published:** 1995-10

**Authors:** C. J. Koch, S. M. Evans, E. M. Lord

**Affiliations:** University of Pennsylvania, Philadelphia 19104-6072, USA.

## Abstract

The present studies were initiated to quantitate the oxygen dependence of bioreductive metabolism-induced binding of EF5, a pentafluorinated derivative of the 2-nitroimidazole, etanidazole. Two different assays were compared: first, radioactive drug incorporation into cell lysates, which provides a direct measure of drug metabolism or uptake; second, monoclonal antibody detection of cellular macromolecular adducts of EF5 after whole cell permeabilisation and fixing. The antibodies (a single clone designated ELK3-51) were conjugated with the fluorescent dye Cy3, with fluorescence determined by fluorescence microscopy and flow cytometry. For the two cell lines tested (V79 Chinese hamster fibroblasts and 9L rat glioma), the oxygen dependence of binding was found to be the same for the two techniques. Using the antibody binding technique, the fluorescence signal was highly reproducible between experiments, resistant to light or chemical bleaching and stable over time following cell or tissue staining. Flow cytometric analysis of cells from rat 9L tumours treated with EF5 in vivo or in vitro showed a distribution of fluorescent signal which was very compatible, on both a relative and absolute basis, with the in vitro results. Our results indicate that immunofluorescent techniques provide a quantitative assay for bioreductive drug adducts, and therefore may be able to measure the absolute oxygen concentration distribution in cell populations and tissues of interest.


British Journal of Cancer (1995) 72, 869-874
© 1995 Stockton Press All rights reserved 0007-0920/95 $12.00

### Oxygen dependence of cellular uptake of EF5 [2-(2-nitro-1H-imidazol-1-yl)-*N*-(2,2,3,3,3-pentafluoropropyl)acetamide]: analysis of drug adducts by fluorescent antibodies *vs* bound radioactivity

CJ Koch[1], SM Evans[2] and EM Lord[3]

[1]*University of Pennsylvania, Radiation Oncology, Philadelphia, PA, 19104-6072;* [2]*University of Pennsylvania, School of Veterinary Medicine, Philadelphia, PA, 19104;* [3]*University of Rochester Cancer Center, Rochester, NY, 14642, USA.*

**Summary** The present studies were initiated to quantitate the oxygen dependence of bioreductive metabolism-induced binding of EF5, a pentafluorinated derivative of the 2-nitroimidazole, etanidazole. Two different assays were compared: first, radioactive drug incorporation into cell lysates, which provides a direct measure of drug metabolism or uptake; second, monoclonal antibody detection of cellular macromolecular adducts of EF5 after whole cell permeabilisation and fixing. The antibodies (a single clone designated ELK3-51) were conjugated with the fluorescent dye Cy3, with fluorescence determined by fluorescence microscopy and flow cytometry. For the two cell lines tested (V79 Chinese hamster fibroblasts and 9L rat glioma), the oxygen dependence of binding was found to be the same for the two techniques. Using the antibody binding technique, the fluorescence signal was highly reproducible between experiments, resistant to light or chemical bleaching and stable over time following cell or tissue staining. Flow cytometric analysis of cells from rat 9L tumours treated with EF5 *in vivo* or *in vitro* showed a distribution of fluorescent signal which was very compatible, on both a relative and absolute basis, with the *in vitro* results. Our results indicate that immunofluorescent techniques provide a quantitative assay for bioreductive drug adducts, and therefore may be able to measure the absolute oxygen concentration distribution in cell populations and tissues of interest.

**Keywords:** fluorescence immunohistochemistry; hypoxic cells; oxygen measurement; oxygen effect; radiation response; bioreductive drugs; 2-(2-nitro-1H-imidazol-1-yl)-*N*-(2,2,3,3,3-pentafluoropropyl)acetamide; 2-nitroimidazole

The bioreductive activation of nitroheterocyclic drugs causes the formation of adducts to cellular macromolecules. We refer to this process as binding. Binding is maximal in the absence of oxygen, and is markedly inhibited as the concentration of oxygen increases; thus, detection of the bound adducts provides an assay signal which increases as the oxygen concentration decreases (Varghese *et al.*, 1976; Chapman, 1979; Koch *et al.*, 1984). Drug binding is therefore an assay for hypoxia, and has been measured in a number of ways: radioactive drug binding allows detection by several different types of radioactivity assays (Varghese *et al.*, 1976; Chapman *et al.*, 1983; Urtasun *et al.*, 1985; Rasey *et al.*, 1987; Parliament *et al.*, 1992), fluorine-containing drug binding allows detection by magnetic resonance spectroscopy or imaging (Raleigh *et al.*, 1984) and antigenic properties of the drug adducts allow secondary detection by antibodies (Raleigh *et al.*, 1987; Hodgkiss *et al.*, 1991; Lord *et al.*, 1993).

The last technique has potential for a very high degree of spatial resolution, and allows assays such as the microscopic distribution of adducts in tissue sections or flow cytometric analysis of individual cells (Hodgkiss *et al.*, 1991). Although both polyclonal and monoclonal antibodies have been developed against various adducts of 2-nitroimidazole drugs (Raleigh *et al.*, 1987; Hodgkiss *et al.*, 1991; Lord *et al.*, 1993), it has not been shown that this technique can provide a quantitative assessment of the number of adducts present. In fact, one might expect this not to be the case since the adducts appear to be formed throughout the entire intracellular environment (Lord *et al.*, 1993), probably at cysteine residues of intracellular proteins (Raleigh and Koch, 1990). It is known that the intracellular concentration of adducts can easily be several hundred micromolar – e.g. after a non-toxic drug exposure of 100 μM for 3 h in nitrogen (Koch, 1990). This concentration corresponds to several hundred million adducts per cell, possibly in very diverse physical–chemical environments. The detection of antigen in such conditions has not previously been studied. Even if one assumed complete access of antibodies to the intracellular environment (e.g. after fixation and permeabilisation) simple calculations suggest that there might not be 'room' to detect all antigen. An antibody concentration of 500 μM would be 7.5% protein (by weight); the preexisting protein content of cells or tissues is typically approximately 20%.

After antibodies have bound to their target antigen, the quantitative detection of the antibodies poses an additional challenge. Use of secondary antibodies or proteins (like avidin) coupled to enzymes causes problems with steric access to the initial adduct sites, prevention of non-specific binding, etc. One is then faced with the necessity of quantitating the amount of product formed (e.g. in enzyme-based assays, any resulting image would have to be analysed by measuring chromophore concentration via absorption measurements). In addition, the use of secondary proteins requires the optimisation of two separate protein binding steps (primary antibody and secondary detection molecule) and the binding of both must be stable during the enzyme reaction. Use of a second protein would only exacerbate the above described protein concentration problems. For these reasons, we have adopted the approach of using a single protein detection system consisting of a monoclonal antibody to the drug adduct, with detection based on the fluorescence of a fluorophore which is chemically coupled to the antibody. In spite of the poor reputation of fluorescence detection as a quantitative technique, this approach had the advantages of: (a) minimising the size of the detecting molecules (since fluorophors have a much lower molecular weight than secondary proteins); (b) allowing the optimisation of binding, including prevention of non-specific binding of a single protein; (c) providing a detection technique which should give a signal directly proportional to the amount of antibody present and (d) allowing the use of flow cytometric analysis techniques to assay the distribution of signal within individual cells of a heterogeneous population.

Correspondence: CJ Koch, Radiation Oncology, University of Pennsylvania, 195 John Morgan Bldg, Philadelphia, PA, 19104-6072, USA
Received 15 December 1994; revised 2 May 1995; accepted 11 May 1995

The 'signal' from an ideal hypoxia detector should have the properties of a consistent maximum signal in the absence of oxygen and the same oxygen dependence of decreasing signal from cell-to-cell or tissue-to-tissue. Much previous work, using cell lines as well as normal and tumour tissue cubes derived from animals and humans, has demonstrated that binding of drugs such as misonidazole fails to demonstrate the ideal properties listed above (Koch, 1990; Koch *et al.*, 1993; Franko and Koch, 1984; Franko *et al.*, 1987; Cobb *et al.*, 1989, 1992). Thus, many different cells and tissues have greatly differing absolute and oxygen-dependent binding of misonidazole. The reasons for this variability in cells are unclear. In contrast, we have found very consistent binding efficiencies for all 2-nitroimidazoles studied using model systems and radiochemical reduction techniques (Raleigh and Koch, 1990). However, these model studies have only been done at very low oxygen concentrations. It seems quite possible however that cell and tissue differences in binding of drugs such as misonidazole may reflect differences in the concentration or type of nitroreductase present (Cobb *et al.*, 1992; Workman, 1992; Joseph *et al.*, 1994). As in the radiation chemical model, little is known about the binding substrates and oxygen dependence of suggested enzymatic nitroreductases. Alternatively, the type, chemical environment or concentration of suitable target binding sites may vary from cell to cell. We showed a tremendous enhancement (100-fold) in binding efficiency in the radiochemical model system by including protein thiols as binding substrates (Raleigh and Koch, 1990). During previous investigations we found two tissue culture cell lines which demonstrated the variability of misonidazole binding to a near maximal extent – WNRE, a subline of V79 Chinese hamster fibroblasts and 9L, a line derived from a rat glioma (Koch, 1990).

We have shown that binding of etanidazole conforms much more closely to the ideal hypoxia detector described above (Koch, 1990; Koch *et al.*, 1993). However, etanidazole is very polar, and tends to form most adducts with acid-soluble cellular molecules (Koch, 1990). High polarity would also prevent etanidazole from distributing evenly to all tissues in animals. Additionally, it does not have a suitable detection scheme. Thus, we have provided an initial characterisation of a pentafluorinated derivative of etanidazole [EF5; 2-(2-nitro-1H-imidazol-1-yl)-*N*-(2,2,3,3,3-pentafluoropropyl)-acetamide] as a hypoxia detector (Lord *et al.*, 1993). Monoclonal antibodies which recognise cellular adducts of the drug were found to be highly specific (Lord *et al.*, 1993). The present study continues this characterisation using the same two cell lines (WNRE and 9L) which previously had been shown to have the most variability in oxygen dependent binding of misonidazole, and using a new monoclonal antibody, ELK3-51, with significantly higher affinity allowing the use of much lower drug concentrations.

### Materials and methods

### *Drug synthesis*

A pentafluorinated derivative [2-(2-nitro-1H-imidazol-1-yl)-*N*-(2,2,3,3,3-pentafluoropropyl)acetamide] of etanidazole, in unlabelled and labelled form ($^{14}$C-2 position; 43 $\mu$Ci mg$^{-1}$) was synthesised by Dr M Tracy and colleagues at Stanford Research International, Palo Alto, CA, USA and is referred to as EF5 in this manuscript.

### *Preparation of cells in tissue culture at defined oxygen conditions*

The cells used were derived from 9L rat glioma (Wong *et al.*, 1991; Evans and Koch, 1994) or the WNRE subline of V79 Chinese hamster fibroblasts (cells obtained in 1981 from Dr JD Chapman, presently of Fox Chase Cancer Center, Philadelphia, PA, USA). The cells were thawed from frozen stock two (WNRE) and four (9L) times per year. Tests were made routinely to ensure that the cultures were free from mycoplasma and other contaminants. The cells were cultured (37°C, 95% air + 5% carbon dioxide, 100% relative humidity) in the exponential phase of growth by transfers at roughly 3.5 day intervals using Eagle's minimal essential medium containing 13% (v/v) of either newborn calf serum (9L) or fetal calf serum (WNRE). Penicillin and streptomycin were also routinely included (all culture solutions from Sigma or Fisher). On the day preceding an experiment, cells were trypsinised and plated onto glass Petri dishes (50 mm diameter; approximately 250 000 cells confined to the central area of the dish followed by overnight incubation at 37°C; Koch, 1984). The dishes were removed from the incubator, cooled to 0–4°C, and their medium was replaced with fresh medium (with or without EF5 as required), first as a rinse (1 ml) which was simply aspirated and then as the actual medium used for the experiment (also 1 ml). Dishes were placed in leakproof aluminium chambers which were connected to a manifold allowing the gas phase of the chambers to be exchanged for the desired oxygen concentration in a series of gas exchanges taking approximately 30 min. The confinement of cells to the central area of the dish, and the use of a small volume of medium allows very rapid equilibration of the gas and liquid phase to improve the control of oxygen concentration (Koch, 1984). After gas exchange, the chambers were immersed in a 37°C water bath, for rapid warming, dried, and transferred to a 37°C warm room. To prevent minor gradients of oxygen or other nutrients or metabolites, the chambers were also shaken gently (1 Hz, 2.5 cm stroke).

In some experiments 9L cells were obtained directly from tumours (see accompanying manuscript). They were dissociated using previously described methods (Howell and Koch, 1980; Evans and Koch, 1994).

### *EF5 binding: radioactivity assay*

Binding of radioactive nitroheterocyclics after incubation under defined experimental conditions was assessed as described previously (Koch *et al.*, 1984, 1993). Briefly, the dishes were removed from the chambers, then medium containing EF5 was replaced with non-drug-containing medium in a series of rinses, the cells were removed from the dishes with trypsin, trypsin was inactivated with serum-containing medium, and the cell number was determined via a particle counter (Coulter). The cells were lysed with 5% trichloroacetic acid and radioactive counts in the acid-soluble *vs* acid-precipitable component were determined with standard liquid scintillation techniques using a Packard 1900 TR counter. The uniformity of the cell samples required no corrections for quenching with $^{14}$C- label. The scintillation fluid was Ecolite (ICN). EF5 binding is equally distributed between acid-soluble and -insoluble fractions under all conditions studied (data not shown) so the total counts were simply combined in the figures.

### *Preparation of monoclonal antibodies*

Monoclonal antibodies were made against radiochemically produced adducts of EF5 and thiol-containing proteins as described previously (Lord *et al.*, 1993). The antibodies used in the present study are derived from a single new clone and are designated as ELK3-51. They have a substantially higher affinity to EF5 and EF5 protein adducts than the antibodies described in our original study (ELK2-4; Lord *et al.*, 1993) (data not shown). The monoclonal antibodies were conjugated with the fluorescent dye, Cy3 (Southwick *et al.*, 1990). This dye is available in a form which reacts with secondary protein amines (Biological Detection Systems, Pittsburgh, PA, USA). The dye–protein ratio was about 4.

### *EF5 Binding: Fluorescence Assay*

Cells were treated as above but radioactive EF5 was not required. After incubation in nitrogen or various levels of oxygen, the dishes were removed from the chambers and cells were removed as above. They were centrifuged out of the inactivated trypsin solution and fixed for 1 h in ice-cold Dulbecco's phosphate-buffered saline (PBS) containing freshly dissolved paraformaldehyde (4%, Sigma P-6148). The pH was adjusted to 7.1–7.4 by the addition of sodium hydroxide (about 400 µl of 1N sodium hydroxide for 200 ml of paraformaldehyde solution). The cells were then rinsed twice in PBS. Non-specific binding was blocked by addition of PBS containing 0.3% Tween 20 (Sigma P-2287), 1.5% albumin, 20% non-fat milk and 5% mouse serum (Jackson Laboratories, 015-000-001) (4°C, overnight). The blocking solution was then removed, the cells rinsed with PBS and antibody added at a concentration of 75 $\mu g\ ml^{-1}$ for 6 h at 4°C. Extensive rinsing (three changes of PBS with 0.3% Tween 20 for 40 min each) was followed by storage of cells in PBS with 1% paraformaldehyde. The exchange of each solution was accomplished by centrifuging cells at 1200 r.p.m. for 12 min, aspirating the supernatant and resuspending in the next solution. Typically, 1–2 million cells were treated in a 2 ml polypropylene centrifuge tube. The cells were blocked and stained in a 75 µl volume, but rinses and fixation used 1 ml volumes. During all but the centrifugation steps cells were maintained in suspension by an oscillatory tipping device (Thermolyne SpeciMix). Stability of the antigen and bound antibody was enhanced by the addition of 1% paraformaldehyde at the end of the procedure. This did not decrease the apparent fluorescence intensity. Cells were either dried onto microscope slides and photographed or analysed by flow cytometry.

The Flow Cytometry Facility at the Cancer Center, University of Pennsylvania, has a FACstar Plus instrument (Becton Dickinson, Mountain View, CA, USA) equipped with a water-cooled 200 mW argon laser. Although the Cy3 dye is optimally excited at 565 nm, the highest available wavelength was 514 nm. Reproducibility of this instrument was excellent on a week to week basis, based on the signal from calibration beads (tetramethyl rhodamine; Flow Cytometry Standards Corporation, NC, USA). A constant voltage of 450 was used for the photodetector tubes.

*Tumour tissue sections (see accompanying manuscript)*

Tumour sections were cut at 14 µm thickness using a Microm HM 505 N cryostat and collected onto poly-L-lysine-coated microscope slides. Staining of the tissue sections was the same as for the whole cells, except that rinses were done by simply moving each slide from container to container.

Fluorescent cells or tissues were photographed using a Nikon epifluorescent microscope. In order to take full advantage of the large dynamic range of the drug–antibody combination with fluorescence photography of cells or tissue sections, it was important to pay attention to all sources of (stray) light in the photographic process. An unexpected problem was 'brought to light' in our continuing attempts to use a sensitive digital camera (Xillix, Vancouver, British Columbia, Canada) rather than the intermediate analogue film. It was found that the microscope's band-pass excitation filter allowed a very substantial infrared (IR) leakage from the mercury lamp; emission filters are usually 'high pass', and even if of the band-pass type, may not specifically exclude IR light. It was easy to identify the IR leakage because the digital camera is very sensitive to such wavelengths, and was clearly responding to something which appeared invisible to human vision. Nevertheless, IR leakage is also very important for conventional film photography. First, it interferes dramatically with the automatic exposure indicator (Nikon, UFX-IIa) which, like the digital camera, is also a solid-state device. Second and more importantly however, the IR leakage can very substantially degrade the image, even using conventional film.

The solution to this problem was to include a multidecade IR cut-off filter (type XF-86; Omega Optical, Brattleboro, VT, USA) in the light path of the emission optics. A convenient location in our microscope was at the base of the camera tube. This type of filter has almost no absorption in the visible spectrum. However, with the IR filter in place, the automatic exposure meter now accurately monitors the actual amount of visible light. In addition to the exposure problem, the IR leakage caused general fogging of the slides. Thus, before the addition of the filter, it was always possible to visualise the separation between adjacent 36 mm fields of an uncut film, even if there was no slide in the field of view. However, it is now often difficult to cut the film because the lowest exposed regions are as dark as the unexposed film between fields.

### Results

Under conditions of extreme hypoxia and over a drug concentration range of 4–100 µM, the 9L cell line showed a 2–3-fold increased rate of $^{14}C$-labelled EF5 binding compared with the WNRE cell line (Figure 1). This result is consistent with a nearly identical oxygen dependence of rate of binding for the two cell lines, considering the 3-fold larger volume of the 9L cell (determined using a Coulter Counter, protein content per cell, or cellular-space measurements; Koch *et al.*, 1989).

Representative data using the antibody detection technique and flow cytometric analysis indicated a contrast ratio of at least 50 between cells incubated in nitrogen *vs* 4% oxygen (Figure 2). The low level of fluorescence seen at relatively high oxygen levels is caused both by low levels of actual drug adducts (see Figure 1) and residual non-specific binding of

**Figure 1** Radioactive drug binding to WNRE and 9L cells as a function of the oxygen concentration in the gas phase of the chambers containing glass dishes with inoculated cells. Open symbols are for 4 µM drug and closed symbols 100 µM drug; (○ and ●), WNRE cells; (□ and ■), 9L cells. Each point represents the average rate of drug uptake, which we have found to be linear with time.

**Figure 2** Representative plots of number of particles (linear scale) *vs* fluorescence intensity (four decade logarithmic scale) for 9L cells incubated with 100 µM EF5 for 3 h at the indicated oxygen partial pressures, then fixed and stained with Cy3-conjugated anti-EF5 monoclonal antibodies (ELK3-51).

the ELK3-51 antibody (see curve for cells incubated without drug). These conclusions arise from the observations that antibody-stained, non-drug treated cells always had a lower level of fluorescence than EF5-treated cells in air, but that cells not stained by the antibody (whether drug-treated or not) had a still lower level of fluorescence (data not shown).

The oxygen dependence of binding was the same using assays based on either radioactive drug uptake or antibody staining (Figures 3 and 4). For measurement of binding of $^{14}$C-labelled EF5 cells were lysed and analysed after about 30 min of rinsing following exposure to drug; however, the antibody measurement technique required extensive time after drug exposure for fixation, blocking, staining and rinsing in solutions containing a mild detergent (0.3% Tween 20). Therefore, it was of interest to measure the loss of radioactive drug metabolites under the same conditions used to effect the antibody staining. We found that more than 50% of the acid precipitable radioactive counts remained with the cells during the antibody staining procedure, while most of the acid soluble counts were lost (data not shown). Thus, although clearly some antigen is lost, this loss appears to be very reproducible and may be associated with drug binding to relatively low molecular weight molecules (work in progress).

Representative flow cytometry of cells obtained from a tumour treated *in vivo* with EF5 (Figure 5) showed a range of binding which was very similar, *on an absolute basis*, to the range observed for cells handled entirely *in vitro*, if allowance is made for the somewhat smaller cells from tumours and the pharmacological decay of drug *in vivo*. One difference was observed however. There was a component of small particles (based on the dot plot of forward *vs* side scatter – see accompanying manuscript) with levels of fluorescence even lower than unstained cells *in vitro* (i.e. particles with fluorescence levels less than 10).

To determine the characteristics of this cell population we disaggregated cells from a tumour in an untreated animal and exposed the resulting cells to 100 μM EF5 under various oxygen conditions *in vitro*. This component did not show an increase in fluorescence under conditions of incubation which would normally enhance binding (i.e. decreasing levels of oxygen with EF5 present), even though the larger cells bound EF5 with the same kinetic characteristics as tissue culture cells (Figure 6). Thus, we believe that this component contains debris and metabolically dead cells.

The presence of this component of particles from disaggregated tumours would explain the larger range of fluorescence intensities found for disaggregated tumour cells treated *in vivo* with EF5 than we have found for tissue culture cells. Additional factors involve the much larger size range of cells from tumours, including red cells and host immune cells. Some of these host cells may be associated with the blood in the tumour at the time of disaggregation, rather than the tumour itself.

**Figure 3** Direct comparison of relative median fluorescence intensity (■) and radioactive drug uptake (O) for WNRE cells incubated with 100 μM EF5 for 3 h at the indicated oxygen partial pressures. Data were normalised to 0.6 under conditions of very low oxygen to move points from axis. The lowest oxygen levels plotted are about 0.01%, which is the *maximum* oxygen in the gas phase of the chambers after the 3 h incubation period.

**Figure 4** Direct comparison of relative median fluorescence intensity (■) and radioactive drug uptake (O) for 9L cells incubated with 100 μM EF5 for 3 h at the indicated oxygen partial pressures. Data normalised as in Figure 3.

**Figure 5** Flow cytometric analysis of cells from 9L tumour (tumour 'A' in accompanying manuscript) incubated *in vivo*, with 100 μM EF5 for 3 h, then fixed, stained and analysed as in Figure 2.

**Figure 6** Flow cytometric analysis of cells from 9L tumour, treated *in vitro* with 100 μM EF5 at various oxygen partial pressures, then fixed, stained and analysed as in Figure 2. Note that one population of cells behaves in nearly identical fashion to cells in tissue culture, but a second population remains at very low fluorescence irrespective of drug and oxygen concentration.

### Discussion

The uniform binding properties of EF5 in cell lines previously shown to exhibit marked heterogeneity of binding of misonidazole suggests that this new drug represents a substantial improvement in the elimination of variability in the oxygen- and cell line-dependent binding by 2-nitroimidazoles (Koch, 1990). Thus, one of the most serious problems with the 2-nitroimidazole binding technique for measuring hypoxia may be eliminated by a suitable choice of compounds. Furthermore, the range of fluorescence intensities at the high values consistent with conditions of very low oxygen are the same on an absolute basis for cells treated for 3 h with 100 μM EF5 either *in vitro* (present manuscript) or *in vivo* (see also accompanying manuscript). This may allow a direct estimation of the range of oxygen concentrations, averaged over the time of drug exposure, for individual cells comprising a tumour.

The stability of the fluorescence signal using the Cy3-labelled ELK3-51 antibody has been found to be excellent, as long as the stained cells are stored in a dilute (1%) paraformaldehyde solution. No degradation in fluorescent signal has been found with storage of stained cells for at least 3 weeks at a temperature of 4°C (data not shown). Previous studies, where the cells were either held in PBS before Ab staining and flow cytometric analysis, or stained and then held in PBS for flow cytometric analysis, showed a slow but continual loss of antigen and/or antibody (data not shown). Storage of cells in PBS with Tween 20 caused a sharp loss in signal after 3–5 days. The signal from tissue sections stained with this antibody and fluorochrome is also exceptionally stable when the slides were stored in cold PBS with 1% paraformaldehyde. We have observed reasonably stable fluorescence over many weeks of storage (based on the photometer associated with the Nikon microscope). The fixative does not have any apparent negative effects on the subsequent analysis of fluorescence. Indeed, this relatively new dye (Southwick *et al.*, 1990) appears to be remarkably free from problems of fading or photobleaching when used in the manner described in this report. Before using simple storage in PBS with dilute fixative we had tried many remedies for preservation of fluorescence, including the use of singlet oxygen or radical scavengers (Valnes and Brandtzaeg, 1985) and various aqueous and non-aqueous coverslip mounting media. Use of mounting media was particularly problematic, sometimes leading to loss of localisation of the antibody and susceptibility to photobleaching. If coverslips are required (for high magnification) we have found that it is best to simply mount the coverslip in the same PBS-paraformaldehyde. Such slides must, of course, be treated extremely carefully to prevent damage to the section and dessication can cause problems unless the slides are stored at 100% relative humidity.

The dynamic range of binding by EF5, as measured by radioactive drug uptake, is very large (factor of at least 50 between nitrogen and air). Thus it is important that the drug adduct-detecting antibodies have very high sensitivity (affinity) and specificity. Clearly, this is the case for ELK3-51, since binding, as detected by flow cytometric analysis, has the same dynamic range and oxygen dependence as does the radioactivity assay. Both assays are sensitive enough to detect the difference in signal between *aerobic* cells with 100 μM drug *vs aerobic* cells without 100 μM drug. However, the immunological method is able to do so on an individual cell basis whereas the radioactivity measurement typically averages the total incorporation of several hundred thousand cells.

For the present limited number of cell lines tested, the oxygen-dependent inhibition of EF5 binding is consistent enough to accurately monitor oxygen over the entire physiological range. In particular, the rate of binding is very similar, on an absolute basis, in the two cell lines (WNRE and 9L) previously shown to have the greatest variability with misonidazole binding. The WNRE line demonstrated half-order kinetics (in other words the rate of binding increased as the square root of misonidazole concentration), and was very sensitive to extremely low oxygen concentrations (Chapman *et al.*, 1983; Koch *et al.*, 1984). The 9L line demonstrated first-order kinetics (rate of binding increased directly with misonidazole concentration) and was not very sensitive to very low oxygen levels (Koch, 1990). These very low oxygen concentrations (below 0.1% oxygen) do not modify the radiation response, so variations in binding in this region of oxygen concentration can produce a highly inaccurate estimate of the radiation resistance. Indeed, extremely hypoxic cells may not be viable *in vivo*, so high binding for extremely *vs* moderately hypoxic cells is undesirable. The details of this relationship remain to be determined however, and may in fact be different for different tumours.

Our current research is directed at testing the hypothesis that the assay of binding of EF5 can predict for the hypoxic fraction, and hence radiation resistance, of individual tumours. The results presented here show that this may be possible using relatively simple flow cytometric techniques. For cells disaggregated from 9L tumours, both the relative and absolute amount of binding were similar to values obtained *in vitro*, if we consider that the cells from tumours were somewhat smaller and had a smaller overall drug exposure (caused by drug distribution and half-life effects). Both factors contribute a factor of about 0.5. Furthermore, the tissue sections from the same tumour showed large regions of very bright binding with varying patterns (see accompanying manuscript). Some but not all of these patterns were consistent with the capillary diffusion model of Thomlinson and Gray (1955). The large dynamic range of the drug–antibody detection scheme shows that there is a continuous distribution of binding throughout the tumour. Although this is what should be expected of the oxygen distribution, it is more common to think of tumours as having an 'aerobic' and 'hypoxic' fraction. Other patterns of drug binding have not previously been observed, namely large fields with high to very high binding throughout. This is not unique to the 9L tumours grown as epigastric tissue isolates, and similar results have been found in subcutaneous 9L tumours as well as FSaII tumours in mice (Evans *et al.*, 1994; Koch *et al.*, 1995).

The present results suggest that binding of EF5 can be assessed quantitatively using immunofluorescent techniques; thus it will be possible to test for inter- and intra-tumour heterogeneity of bound EF5 using simple biopsy techniques and flow cytometric analysis. The capability of using flow cytometry techniques will then allow the simultaneous assay of many other important tumour/host cell properties. In addition, the use of fluorescence immunohistochemical techniques allows for the very rapid analysis of the distribution of EF5 binding in any normal/tumour tissue of interest. Quantitation of the two-dimensional change in fluorescence intensity appears very possible, requiring only a reliable light source and/or fluorescence standard coupled with the digital analysis of fluorescence intensity. Current work is moving from the use of a slide-film intermediate to direct digital image acquisition (Xillix Technologies, Vancouver, British Columbia, Canada). Thus, we anticipate that the direct test of the above-mentioned hypothesis is within reach.

**Acknowledgements**

This work was supported by grants from the American Cancer Society, BE187C (CJK), and National Institutes of Health CA-56679 (SME, CJK), CA-28332 (EML).

### References

CHAPMAN JD. (1979). Hypoxic sensitizers – implications for radiation therapy. *New Engl. J. Med.*, **301**, 1429–1432.

CHAPMAN JD, BAER K AND LEE J. (1983). Characteristics of the metabolism-induced binding of misonidazole to hypoxic mammalian cells. *Cancer Res.*, **43**, 1523–1528.

COBB LM, NOLAN J AND O'NEILL P. (1989). Microscopic distribution of misonidazole in mouse tissues. *Br. J. Cancer*, **59**, 12–16.

COBB LM, NOLAN J AND HACKER T. (1992). Retention of misonidazole in normal and malignant tissues: interplay of hypoxia and reductases. *Int. J. Radiat. Oncol. Biol. Phys.*, **22**, 655–659.

EVANS SM AND KOCH CJ. (1994). Characterization of the 9L glioma as a tissue isolated epigastric implant. *Radiat. Oncol. Invest*, **2**, 134–143.

EVANS SM, KOCH CJ, JOINER B, JENKINS WT, LAUGHLIN KM AND LORD EM. (1995). 2-Nitroimidazole binding for identification of hypoxic cell fraction in cells and tissues of epigastric 9L tumours. *Br. J. Cancer*, **72**, 888–895.

FRANKO AJ AND KOCH CJ. (1984). Binding of misonidazole to V79 spheroids and fragments of Dunning rat prostate and human colon carcinoma in vitro: diffusion of oxygen and reactive metabolites. *Int. J. Radiat. Oncol. Biol. Phys.*, **10**, 1333–1337.

FRANKO AJ, KOCH CJ, GARRECHT BM, SHARPLIN J AND HOWORKO J. (1987). Oxygen dependence of binding of misonidazole to rodent and human tumours in vitro. *Cancer Res.*, **47**, 5367–5376.

HODGKISS RJ, JONES G, LONG A, PARRICK J, SMITH KA AND STRATFORD MRL. (1991). Flow cytometric evaluation of hypoxic cells in solid experimental tumours using fluorescence immunodetection. *Br. J. Cancer*, **63**, 119–125.

HOWELL RL AND KOCH CJ. (1980). The disaggregation, separation and identification of cells from irradiated and unirradiated EMT6 mouse tumors. *Int. J. Radiat. Oncol. Biol. Phys.*, **6**, 311–318.

JOSEPH P, JAISWAL K, STOBBE C AND CHAPMAN JD. (1994). The role of specific reductases in the intracellular activation and binding of 2-nitroimidazoles. *Int. J. Radiat. Oncol. Biol. Phys.*, **29**, 351–355.

KOCH CJ. (1984). A thin-film culturing technique allowing rapid gas-liquid equilibration (6 seconds) with no toxicity to mammalian cells. *Radiat. Res.*, **97**, 434–442.

KOCH CJ. (1990). The reductive activation of nitroimidazoles; modification by oxygen and other redox-active molecules in cellular systems. In *Selective Activation of Drugs by Redox Processes*, Adams GE, Breccia A, Fielden EM and Wardman P. (eds) pp. 237–247. Plenum Press: New York.

KOCH CJ, STOBBE CC AND BAER KA. (1984). Metabolism induced binding of $^{14}$C-misonidazole to hypoxic cells: kinetic dependence on oxygen concentration and misonidazole concentration. *Int. J. Radiat. Oncol. Biol. Phys.*, **10**, 1327–1332.

KOCH CJ, STOBBE CC AND HETTIARATCHI P. (1989). Combined radiation-protective and radiation-sensitizing agents: IV) Measurement of intracellular protector concentrations. *Int. J. Radiat. Oncol. Biol. Phys.*, **16**, 1025–1027.

KOCH CJ, GIANDOMENICO AR AND LEE IYENGAR CW. (1993). Bioreductive metabolism of AF-2 [2(2-furyl)-3-(5-nitro-2-furyl)-acrylamide] combined with 2-nitroimidazole radiosensitizing agents. *Biochem. Pharmacol.*, **46**, 1029–1036.

KOCH CJ, EVANS SM AND LORD EM. (1995). Comment on the hypothesis that hyperthermia facilitates reoxygenation. *Int. J. Hyperthermia*, **11**, 447–450.

LORD EM, HARWELL L AND KOCH CJ. (1993). Detection of hypoxic cells by monoclonal antibody recognizing 2-nitroimidazole adducts. *Cancer Res.*, **53**, 5271–5276.

PARLIAMENT MB, CHAPMAN JD, URTASUN RC, MCEWAN AJ, GOLBERG L, MERCER JR, MANNAN RH AND WIEBE LI. (1992). Non-invasive assessment of human tumour hypoxia with l-iodoazomycin arabinoside: preliminary report of a clinical study. *Br. J. Cancer*, **65**, 90–95.

RALEIGH JA AND KOCH CJ. (1990). The importance of thiols in the reductive binding of 2-nitroimidazoles to macromolecules. *Biochem. Pharmacol.*, **40**, 2457–2464.

RALEIGH JA, FRANKO AJ, TREIBER EO, LUNT JA AND ALLEN PS. (1984). Covalent binding of a fluorinated 2-nitroimidazole to EMT-6 tumours in Balb/c mice: detection by F-19 nuclear magnetic resonance at 2.35 T. *Int. J. Radiat. Oncol. Biol. Phys.*, **10**, 1337–1340.

RALEIGH JA, MILLER GG, FRANKO AJ, KOCH CJ, FUCIARELLI AF AND KELLEY DA. (1987). Fluorescence immunohistochemical detection of hypoxic cells in spheroids and tumours. *Br. J. Cancer*, **56**, 395–400.

RASEY JS, GRUNBAUM Z, MAGEE S, NELSON NJ, OLIVE PL, DURAND RE AND KROHN KA. (1987). Characterization of radiolabelled fluoromisonidazole as a probe for hypoxic cells. *Radiat. Res.*, **111**, 292–304.

SOUTHWICK PL, ERNST LA, TAURIELLO EW, PARKER SR, MUJUMDAR RB, MUJUMBDAR SR, CLEVER HA AND WAGGONER AS. (1990). Cyanine dye labeling reagents - carboxymethylindocyanine succinimidyl esters. *Cytometry*, **11**, 418–430.

THOMLINSON RH AND GRAY LH. (1955). The histological structure of some human lung cancers and the possible implications for radiotherapy. *Br. J. Cancer*, **9**, 539–579.

URTASUN RC, KOCH CJ, FRANKO AJ, RALEIGH JA AND CHAPMAN JD. (1985). A novel technique for measuring human tissue hypoxia at the cellular level. *Br. J. Cancer*, **54**, 453–457.

VALNES K AND BRANDTZAEG P. (1985). Retardation of immunofluorescence fading during microscopy. *J. Histochem. Cytochem.*, **33**, 755–761.

VARGHESE AJ, GULYAS S AND MOHINDRA JK. (1976). Hypoxia-dependent reduction of 1-(2-nitro-1-imidazolyl)-3-methoxy-2-propanol by Chinese hamster ovary cells and KHT tumor cells in vitro and in vivo. *Cancer Res.*, **36**, 3761–3765.

WONG K-H, KOCH CJ, WALLEN CA AND WHEELER KT. (1991). Pharmacokinetics and cytotoxicity of RSU-1069 in subcutaneous 9L tumours under oxic and hypoxic conditions. *Br. J. Cancer*, **63**, 484–488.

WORKMAN P. (1992). Bioreductive mechanisms. (Keynote address) *Int. J. Radiat. Oncol. Biol. Phys.*, **22**, 631–637.